# Imidazole Derivative As a Novel Translation Inhibitor

**DOI:** 10.32607/actanaturae.11654

**Published:** 2022

**Authors:** D. A. Lukianov, V. S. Buev, Y. A. Ivanenkov, V. G. Kartsev, D. A. Skvortsov, I. A. Osterman, P. V. Sergiev

**Affiliations:** Skolkovo Institute of Science and Technology, Center of Life Sciences, Skolkovo, 143028 Russia; Lomonosov Moscow State University, Faculty of Bioengineering and Bioinformatics, Moscow, 119991 Russia; Institute of Biochemistry and Genetics Russian Academy of Science (IBG RAS), Ufa Scientific Centre, Ufa, 450054 Russia; The Federal State Unitary Enterprise Dukhov Automatics Research Institute, Moscow, 127055 Russia; InterBioScreen Ltd, Chernogolovka, 142432 Russia; Lomonosov Moscow State University, Chemistry Department, Moscow, 119991 Russia; Higher School of Economics, Faculty of biology and biotechnologies, Moscow, 101000 Russia; Sirius University of Science and Technology, Genetics and Life Sciences Research Center, Sochi, 354340 Russia; Lomonosov Moscow State University, Institute of functional genomics, Moscow, 119991 Russia

**Keywords:** translation, bacteria, translation inhibitor, compounds with antibiotic activity, imidazole derivatives

## Abstract

Searching for novel compounds with antibiotic activity and understanding their mechanism of action is extremely important. The ribosome is one of the main targets for antibiotics in bacterial cells. Even if the molecule does not suit the clinical application for whatever reasons, an investigation of its mechanism of action can deepen our understanding of the ribosome function. Such data can inform us on how the already used translational inhibitors can be modified. In this study, we demonstrate that 1-(2-oxo-2-((4-phenoxyphenyl)

## INTRODUCTION

The coronavirus pandemic has highlighted the problem of human vulnerability to pathogens. The outlook for the expansion of antibiotic resistance in bacteria is also unfavorable. According to the Organization for Economic Cooperation and Development (OECD), approximately 17% of infectious diseases in OECD member countries were rooted in antibiotic resistance of bacteria in 2015. In the Russian Federation, the percentage of such diseases exceeds 40% [1]. As of 2016, ~700,000 people die each year from infections caused by antibiotic-resistant bacteria. According to a prediction published in 2016, the number of deaths caused by resistant bacteria may be as high as 10 million people by 2050 [2]. Hence, modern science currently faces the challenge of coming up with novel antibiotics.

The protein synthesis occurring on ribosomes is the vital process through which the genetic information in the mRNA is translated into the amino acid sequence of a protein. The bacterial ribosome consists of three ribosomal RNAs (16S, 23S, and 5S) and more than 50 proteins, forming two subunits; the small, 30S, subunit and the large, 50S, one; they combine to form the 70S ribosome. Each of these subunits and the exit tunnel through which the newly synthesized peptide is released are targets for a large number of antibiotics [3]. Thus, tetracycline [4], streptomycin [5], pactamycin [6], and amicoumacin A [6] bind to the small ribosomal subunit. The structure of the complex formed between the ribosome and each of these antibiotics has been determined. Chloramphenicol [7], clindamycin [8], and blasticidin C [9] can bind to the large ribosomal subunit, thus leading to protein synthesis arrest. The antibiotics tetracenomycin X [10], klebsazolicin [11], and erythromycin [7] inhibit peptide release from the ribosome. According to published data, more than half of all drugs used to treat infections belong to the class of protein synthesis inhibitors [12]. Therefore, understanding the ribosome function can be crucial both in the search for novel drugs and in improving the ones that are already in our possession. We have described a novel translation inhibitor, and understanding of its mechanism of action can be highly valuable both in fundamental science and, after we master it, in the real-world healthcare system.

## EXPERIMENTAL

**Application of a dual reporter system for analyzing the mechanism of action of antibiotics**

**Fig. 1:**
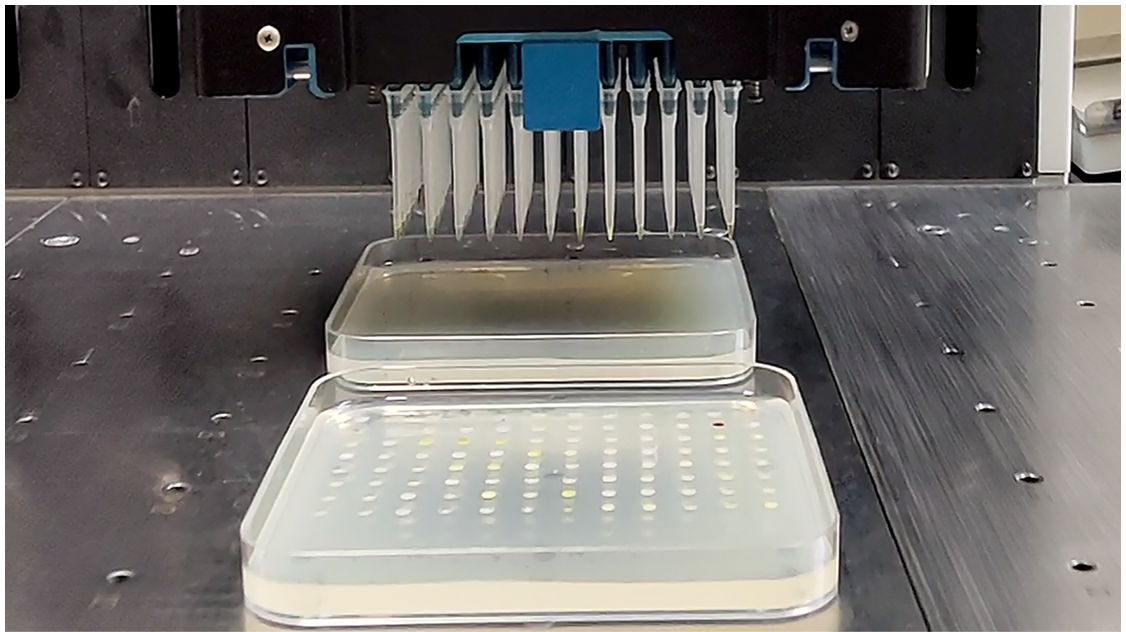
Transferring 96 individual molecules onto the cell lawn

The mechanism of antibiotic action was studied using the pDualrep2 reporter system [13]. For conducting the assay, an overnight culture of *Escherichia coli *JW5503 cells [14] frozen in 50% glycerol was diluted tenfold in LB liquid broth and inoculated in Petri dishes containing 1.5% LB agar and ampicillin (50 µg/mL) to obtain a bacterial lawn. The culture dishes were dried, and 96 samples of different molecules (*Fig. 1*) were applied onto their surface using a Janus robotic workstation (Perkin Elmer, USA). Before their application, the compounds were dissolved in dimethyl sulfoxide (DMSO, PharmaMed, Russia) to a concentration of 20 mg/mL. Each compound (30 μg) was applied. The culture dishes, containing cells, were then incubated for 18 h at 37°C. To visualize the results, the culture dishes were scanned using a ChemiDoc imaging system (Bio-Rad, USA) in Cy3 (for TurboRFP detection) and Cy5 channels (for Katushka2S detection).

**Measuring the minimum inhibitory concentration**

The minimum inhibitory concentration was measured in 96-well plates. Plate rows (1–11) were filled with a *E. coli *(JW5503) cell suspension obtained by diluting the overnight culture 200-fold. 200 μL of the cells was added to the first row, and 100 μL of the cells was added to the subsequent rows. The last plate row (row 12) was filled with the LB culture medium without cells to control for the validity of the experiment.

The test compound (2 µL; concentration, 20 mg/mL) was added to the cells of row 1, followed by a series of twofold dilutions in the subsequent rows (up to row 10). For this purpose, 100 µL of the mixture was transferred from the first well to the second one using an eight-channel pipette, mixed, and the procedure was repeated up to row 10. Erythromycin (2 µL; concentration, 5 mg/mL) was added to one of the rows of each plate instead of the test substance as a control. The plates were then subjected to aeration incubation at 37°C overnight at 200 rpm. Cell concentration was estimated according to the absorbance (*A*600). The measurements were performed on a Victor X5 2030 plate reader (Perkin Elmer).

The lowest concentration of the test compound that completely inhibited bacterial growth was considered the minimum inhibitory concentration.

**Cytotoxicity test**

The cytotoxicity of the test compound was verified using the MTT method (3-(4,5-dimethylthiazol- 2-yl)-2,5-diphenyl-tetrazolium bromide) [15], with the certain modifications. Some 2,500 cells per well for MCF7, HEK293T, and A549 cell lines or 4,000 cells per well for the VA-13 cell line were inoculated into 135 µL of the DMEM-F12 medium (Gibco, USA) in a 96-well plate and incubated in a 5% CO_2_ incubator for the first 16 h without any treatment. Next, 15 µL of a mixture of the medium and a solution of the test compound in DMSO (final DMSO concentrations in the media were ≤1%) were added to the cells; the cells were treated for 72 h in the presence of the test compounds in eight dilutions (100 ng/mL – 200 µg/mL); three replicates were made for each dilution; doxorubicin was used as a control substance. MTT was then added to the cells (OJSC PanEco, Russia) to a final concentration of 0.5 g/L (a supernatant fluid diluted in PBS tenfold was used), and the cells were incubated for 2 h in an incubator in an atmosphere of 5% CO_2_ at 37°C. The MTT solution was then removed, and 140 µL of DMSO (OJSC Pharmamed, Russia) was added. The plates were shaken (80 rpm) to let formazan dissolve. Absorbance was measured using a Victor X5 2030 plate reader (Perkin Elmer) at 565 nm (for measuring formazan concentration). The results were used to plot the dose–response relationships and assess the IC_50_ value (GraphPad Software, Inc., USA).

***In vitro *translation**

The ability of the tested compound to inhibit translation was determined using a *E. coli *S30 Extract System for Linear Templates kit (Promega, USA).

The reaction was conducted in 5 µL of the mixture having the following composition: 2 µL of S30 Premix, 1.5 µL of S30 from the *E. coli *cell extract, 0.5 µL of the amino acid mixture (concentration of each amino acid, 1 mM), 0.5 µL of mRNA (Fluc 200 ng/µL), 0.2 mM *D*-luciferin, 0.1 µL of the RiboLock RNase inhibitor, and 0.5 µL of the test compound. The reaction mixture (except for mRNA) was pre-mixed on ice and then incubated at room temperature for 5 min to give the antibiotic ample time to bind to the ribosome before the initiator complex assembly; the mixture was then returned on ice, and the template was added.

Translation was carried out for 20 min at 37°C. The signal was then detected on a Victor X5 2030 plate reader (Perkin Elmer).

**Toeprinting assay**

The toeprinting assay was conducted according to the protocol described by Orelle et al. [16].

At the first stage, the primers were labeled with [γ-32P]ATP polynucleotide kinase (ThermoFisher, USA) according to the manufacturer’s protocol. Next, *in vitro *translation of the short-model mRNA was performed using a PURExpress® *In Vitro* Protein Synthesis Kit (New England Biolabs, USA). The reaction mixture (volume, 5 µL) contained 2 µL of solution A, 1 µL of solution B, 0.2 µL of RiboLock (ThermoFisher), 0.5 µL of the test compound, 0.5 µL of DNA template (0.2 mmol/µL), and 0.5 µL of the radiolabeled primer. The mixture was incubated at 37°C for 20 min, and 1 µL of the reverse transcription mix from the Titan One Tube RT-PCR System kit (Roche, Switzerland) was added. Reverse transcription was conducted for 15 min at 37°C. The reaction was stopped by adding 1 µL of 10 M NaOH, followed by incubation at 37°C for 15 min. The neutralization was performed by adding 1 µL of 10 N HCl. Next, 200 µL of the resuspension buffer as added.

The resulting samples were purified using a QIAquick PCR purification kit (Qiagen, Germany).

The sequence mixtures were prepared using a USB® Thermo Sequenase Cycle Sequencing Kit (Affymetrix, USA) according to the manufacturer’s protocol.

Electrophoresis was carried out in 6% polyacrylamide gel (60 × 40 × 0.03 cm) containing 19% acrylamide, 1% N,N’-methylenebisacrylamide, and 7 M urea in TBE buffer for 2–3 h. The specimens and products of the sequencing reactions (2 and 1.5 µL, respectively) were applied onto the gel.

The gel was transferred onto 3-mm paper, dried, and exposed to a sensory screen for 18 h. The screen was scanned using a Typhoon FLA 9500 Biomolecular Imager (GE Healthcare, USA).

The RST1 template for this experiment was obtained by PCR amplification using a Taq-DNApolymerase kit (ThermoFisher), according to the standard protocol. The template sequence was as follows: ACTAATACGACTCACTATAGGGCTTAAGTATAAGGAGGAAAACATATGTATTGGGTAACCTCACGTCAGCCGAATATGCTGAAAATCCATGGCTTCGAAGACTGCGCCTAATAATAA TAAAAAAAGTGATAGAATTCTATCGTTAATAAGCAAAATTCATTATAAC. The forward primer GTAAAACGACGGCCAGT, reverse primer CAGGAAACAGCTATGAC, and primer for reverse transcription GGTTATAATGAATTTTGCTTATTAAC were used.

## RESULTS AND DISCUSSION

**High-throughput screening for compounds exhibiting antimicrobial activity**

**Fig. 2:**
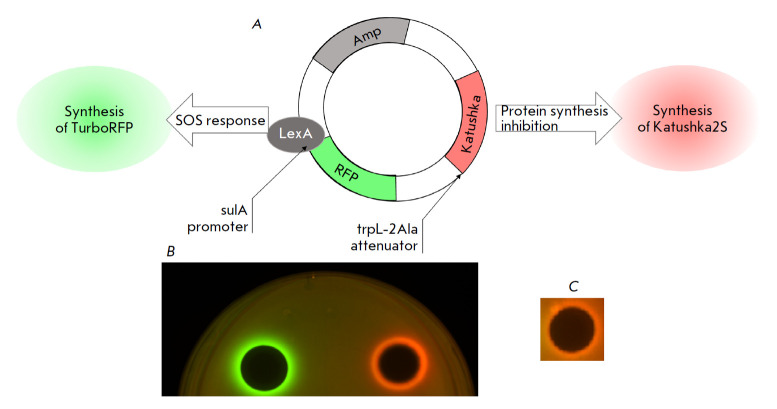
(*A*) – the composition of the pDualrep2 reporter plasmid. (*B*) – induction of a two-color dual-reporter system sensitive to inhibitors of ribosome progression or DNA replication. Drops of erythromycin (right-hand side, 2 μg) and levofloxacin (left-hand side, 0.05 μg) were placed on the surface of an agar plate containing *E. coli *JW5503 cells transformed with the pDualrep2 plasmid. Expression of Katushka2S (red) is induced by translation inhibitors, whereas RFP expression (green) is induced upon DNA damage. (*C*) – induction of a two-color dual-reporter system induced by 1-(2-oxo-2-((4-phenoxyphenyl)amino)ethyl)-3-(*p*-tolyl)-6,7-dihydro-5H-pyrrolo[1,2-a]imidazol-1-ium chloride (30 μg)

An *E. coli *JW5503 strain with *tolC *deletion [14] transformed with plasmid pDualrep2 [13] was used for screening for compounds exhibiting antimicrobial activity (*Fig. 2A*). The *tolC *gene is responsible for the synthesis of the AcrAB-TolC component of the efflux system, and its deletion renders cells more sensitive to the test compounds [17]. The reporter system functions according to the following principle: if a compound inhibits protein synthesis in the cell, it results in ribosomal stalling on the modified tryptophan operon sequence (trpL-2-Ala), which induces the synthesis of the far-red fluorescent protein Katushka2S (*Fig. 2B*, red pseudo-color). DNA damage-inducing compounds elicit the SOS response in the cell, thus causing dissociation of the LexA repressor protein from the *sulA *promoter and initiation of the expression of the gene encoding the TurboRFP red fluorescent protein (*Fig. 2B*, green pseudo-color).

**Fig. 3:**
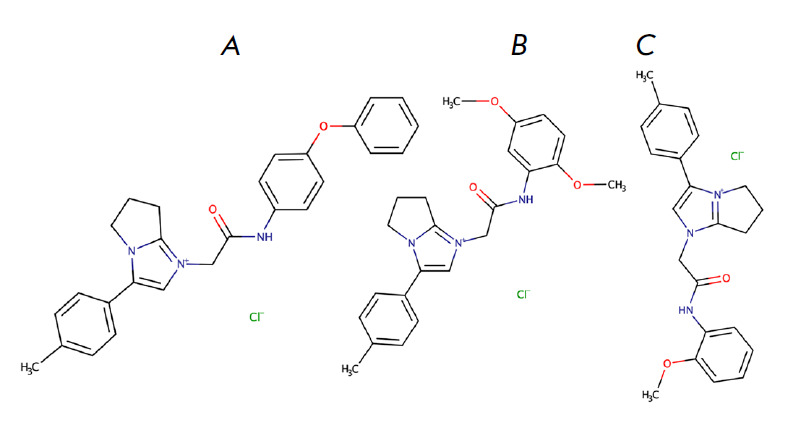
(*A*) – structural formula of the active compound 1-(2-oxo-2-((4-phenoxyphenyl)amino)ethyl)-3-(*p*-tolyl)- 6,7-dihydro-5H-pyrrolo[1,2-a]imidazol-1-ium chloride (STOCK4S-33513). (*B*) – structural formula of the active compound analog 1-(2-((2,5-dimethoxyphenyl)amino)-2- oxoethyl)-3-(*p*-tolyl)-6,7-dihydro-5H-pyrrolo[1,2-a]imidazol- 1-ium chloride (STOCK4S-37310). (*C*) – structural formula of the active compound analog 1-(2-((2-methoxyphenyl) amino)-2-oxoethyl)-3-(*p*-tolyl)-6,7-dihydro-5Hpyrrolo[ 1,2-a]imidazol-1-ium chloride (STOCK4S-72264)

During the high-throughput screening libraries of the chemical compounds provided by InterBioScreen Ltd., among the compounds with antimicrobial activity, we found a molecule that both inhibited growth of the *E. coli *strain JW5503 transformed with plasmid pDualrep2 and induced the expression of the *Katushka2S *gene that is typical of translation inhibitors (*Fig. 2C*). The formula of this molecule, 1-(2-oxo-2-((4-phenoxyphenyl)amino)ethyl)-3- (*p*-tolyl)-6,7-dihydro-5H-pyrrolo[1,2-a]imidazol-1-ium chloride, is shown in *Fig. 3A* (STOCK4S-33513). During screening, two analogs of this molecule were tested: 1-(2-((2,5-dimethoxyphenyl)amino)-2- oxoethyl)-3-(*p*-tolyl)-6,7-dihydro-5H-pyrrolo[1,2-a] imidazol-1-ium chloride (STOCK4S-37310, *Fig. 3B*) and 1-(2-((2-methoxyphenyl)amino)-2-oxoethyl)-3- (*p*-tolyl)-6,7-dihydro-5H-pyrrolo[1,2-a]imidazol-1-ium chloride (STOCK4S-72264, *Fig. 3C*). These molecules did not yield growth inhibition zones for the* E. coli *JW5503 strain transformed with plasmid pDualrep2 in the solid agar medium test; therefore, further experiments were conducted using exclusively 1-(2-oxo-2-((4-phenoxyphenyl)amino)ethyl)-3- (*p*-tolyl)-6,7-dihydro-5H-pyrrolo[1,2-a]imidazol-1-ium chloride.

This compound was inactive in the test for the* E. coli *BW25113 strain transformed with the reporter plasmid.

**Measuring the minimum inhibitory concentration**

The minimum inhibitory concentration was measured using the serial dilution method for the *E. coli* JW5503 strain with *tolC *deletion [14]. The minimum inhibitory concentration of 1-(2-oxo-2-((4-phenoxyphenyl) amino)ethyl)-3-(*p*-tolyl)-6,7-dihydro-5Hpyrrolo[ 1,2-a]imidazol-1-ium chloride is 3.1 µg/mL. It is comparable with that of erythromycin, a natural protein synthesis inhibitor whose minimum inhibitory concentration for this strain was 3.1 µg/mL.

**Measuring cytotoxicity in eukaryotic cells**

**Table:** The MTT assay results for 1-(2-oxo-2-((4-phenoxyphenyl) amino)ethyl)-3-(p-tolyl)-6,7-dihydro-5H-pyrrolo[1,2-a] imidazol-1-ium chloride

Cell line	Concentration*, µg/mL
HEK293T	0.2 ± 0.1
MCF7	1.8 ± 0.5
A549	0.5 ± 0.1
Va-13	0.4 ± 0.2

^*^Concentration of the test compound toxic to cells, μg/mL.

The toxicity of this compound was tested in several human cell cultures using the MTT assay. Unfortunately, it was found to be more toxic to human cells compared to bacterial ones; so, this compound cannot be used as a drug, at least in the tested form. The data are summarized in *Table*.

**Translation in the cell-free system**

**Fig. 4:**
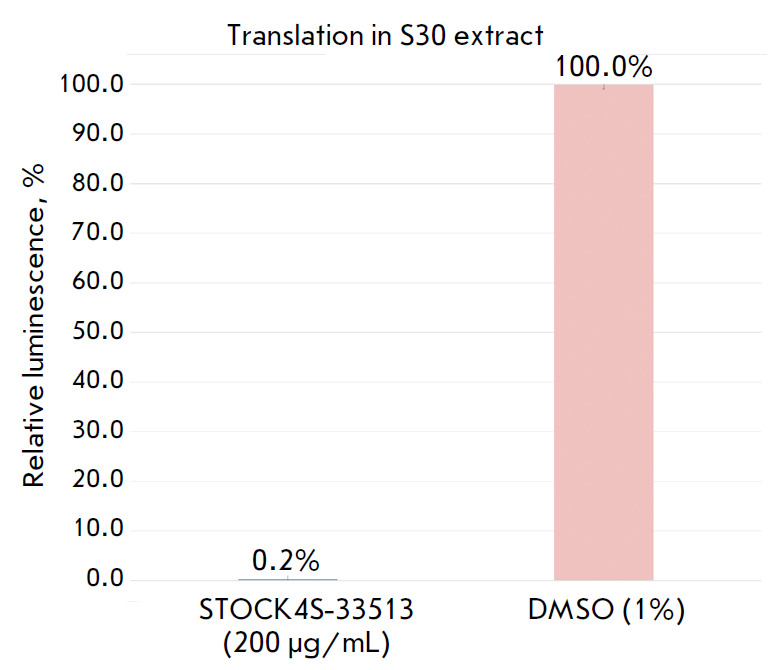
Protein synthesis inhibition with 200 μg/mL of 1-(2-oxo-2-((4-phenoxyphenyl)amino)ethyl)-3-(*p*-tolyl)- 6,7-dihydro-5H-pyrrolo[1,2-a]imidazol-1-ium chloride using an *in vitro *cell-free translation system. The activity of luciferase synthesised using an *in vitro *cell-free translation system without translation inhibitors is taken as 100%

The translation reaction in the cell-free system was carried out using a *E. coli *S30 Extract System for Linear Templates kit (Promega). Synthesis of firefly luciferase in this experiment was determined using the reaction of luciferin oxidation to oxyluciferin. If the reaction mixture contains a translation inhibitor, luciferase is not synthesized and luciferin is not degraded. The results of each experiment were normalized with respect to the added solvent (dimethyl sulfoxide), whose volume was identical to that of the test compound. The data for 1-(2-oxo-2-((4-phenoxyphenyl)amino)ethyl)-3-(*p*-tolyl)- 6,7-dihydro-5H-pyrrolo[1,2-a]imidazol-1-ium chloride (STOCK4S-33513) are presented in *Fig. 4*.

According to the results, it appears fair to say that the compound 1-(2-oxo-2-((4-phenoxyphenyl)amino) ethyl)-3-(*p*-tolyl)-6,7-dihydro-5H-pyrrolo[1,2-a]imidazol- 1-ium chloride (STOCK4S-33513) is a translation inhibitor.

**Analysis of the ribosome stall sites**

**Fig. 5:**
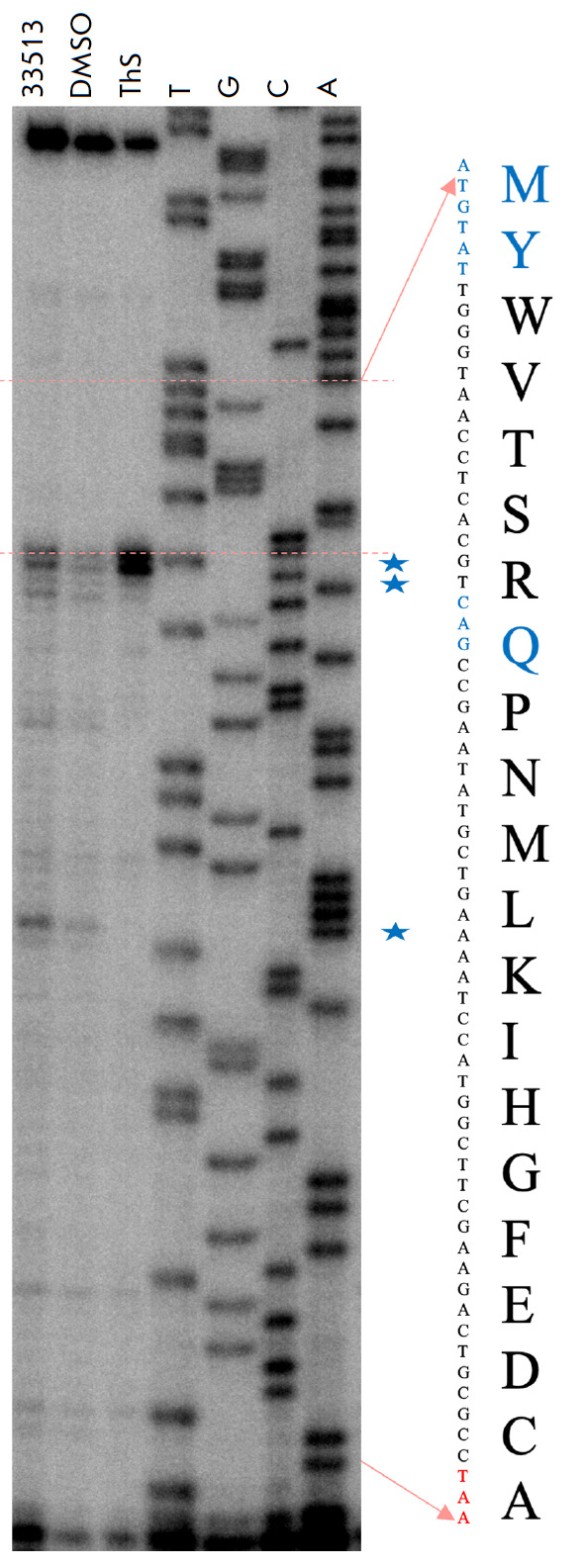
The scheme of toeprinting assay on the RST1 template: lane 1 (STOCK4S-33513) corresponds to the *in vitro *cell-free translation system supplemented with 200 μg/mL 1-(2-oxo-2-((4-phenoxyphenyl)amino)ethyl)-3- (*p*-tolyl)-6,7-dihydro-5H-pyrrolo[1,2-a]imidazol-1-ium chloride; lane 2 (DMSO) corresponds to the negative control (1% DMSO); lane 3 (Ths) corresponds to 50 μM thiostrepton (Ths inhibits translation at the start codon [18]); T, G, A, C are the lanes corresponding to sequencing reactions with serial stops at the corresponding nucleotides

Not only does the toeprinting assay allow one to verify whether a compound inhibits protein synthesis, or not, but it also makes it possible to hypothesize regarding the stage at which translation was stalled. The working principle of the method is as follows: in a cell-free system that is based on individually isolated translation components, a short peptide is synthesized in the presence of the test compound. A radiolabeled primer (complementary to the 3’-terminus of mRNA), RNA-dependent DNA polymerase, and 2’-deoxynucleoside-5’-triphosphates are added to the reaction mixture. Next, template RNA-directed primer extension takes place until RNA-dependent DNA polymerase either meets the ribosome or reaches the template end. If a protein synthesis inhibitor is added to the mixture, the ribosome will stall on the template and will not allow RNA-dependent DNA polymerase to reach the end of the template; so, the cDNA fragment will be short. The exact length of the cDNA fragment and the ribosome stall site on mRNA can be calculated according to the RNA sequence and position of the reverse transcription product in the gel with respect to the Sanger sequencing products being separated in the respective gel lanes. In a typical experimental run, we also compared the sites of ribosome stalling induced by the novel and already known translation inhibitors. The distance between the first nucleotide of the P site of the ribosome blocked on mRNA and the last synthesized cDNA nucleotide is 16 nucleotides long. It is convenient to use the thiostrepton antibiotic for comparison as it is known to induce ribosome stalling at the first translation step, right when the start codon AUG resides at the ribosomal P site. Based on these data, we have performed computations for the codons residing at the P site at the instant of ribosomal stalling (*Fig. 5*). These codons were 1-AUG (M), 2-UAU (Y), and 8-CAG (Q). However, in the control experiment without the DMSO antibiotic added, one can see the same short pauses (but less pronounced) at the same spots. Therefore, a hypothesis can be put forward that this translation inhibitor can affect the kinetics of protein synthesis at mRNA regions that are difficult for the ribosome to traverse.

## CONCLUSIONS

We have investigated a novel inhibitor of bacterial translation, 1-(2-oxo-2-((4-phenoxyphenyl)amino) ethyl)-3-(*p*-tolyl)-6,7-dihydro-5H-pyrrolo[1,2-a]imidazol- 1-ium chloride, retrieved from the chemical library. This compound was shown to induce the reporter system and act as an *in vivo *translation inhibitor. It was revealed that it can inhibit *in vitro* translation and potentiate ribosomal stalling during the synthesis of small peptides. Although this compound is highly toxic to human cells and, thus, cannot be used as a drug, a more thorough examination of this molecule may provide a deeper insight into the functioning of such an important molecular machine as ribosome.
